# S215. THE APROSODY OF SCHIZOPHRENIA: COMPUTATIONALLY DERIVED ACOUSTIC PHONETIC UNDERPINNINGS OF MONOTONE SPEECH

**DOI:** 10.1093/schbul/sby018.1002

**Published:** 2018-04-01

**Authors:** Michael Compton, Luca Pauselli, Anya Lunden, Sean Cleary, Yazeed Alolayan, Brooke Halpern, Beth Broussard, Anthony Crisafio, Leslie Capulong, Pierfrancesco Balducci, Francesco Bernardini, Michael Covington

**Affiliations:** 1 College of Physicians and Surgeons, Columbia University; 2 College of Physicians and Surgeons, Columbia University, New York State Psychiatric Institute; 3 College of William and Mary; 4 The George Washington University; 5 Case Western Reserve University; 6 Lenox Hill Hospital; 7 Università Degli Studi Di Perugia; 8 University of Georgia

## Abstract

**Background:**

Acoustic phonetics methods are useful in examining some symptoms of schizophrenia; we used such methods to understand the underpinnings of aprosody. We hypothesized that compared to controls and patients without clinically rated aprosody, patients with aprosody would exhibit reduced variability in: pitch, jaw/mouth opening and tongue height (formant F1), tongue front/back position and/or lip rounding (F2), and intensity/loudness.

**Methods:**

Audio-recorded speech was obtained from 98 patients (including 25 with clinically rated aprosody and 29 without) and 102 unaffected controls using five tasks: one pertaining to describing a drawing (Task 1), two based on spontaneous speech elicited through a question (Tasks 2 and 3), and two based on reading prose (Tasks 4 and 5). We compared the three groups (patients with aprosody, patients without aprosody, and controls) in terms of variation in pitch, formants F1 and F2, and intensity/loudness.

**Results:**

Phonetic values were generally highly correlated across the five speech tasks. Regarding pitch variation, in unadjusted tests, patients with aprosody differed significantly from controls in Tasks 3 and 4; for Task 5, the difference was statistically significant in both unadjusted tests and those adjusted for sociodemographics. For the standard deviation (SD) of F1, the expected pattern was observed in the two reading tasks in adjusted tests (lower values for patients with aprosody, intermediate values for patients without aprosody and higher values for controls). Regarding SD of F2, patients with aprosody had lower values than controls in unadjusted tests across all tasks; in adjusted tests the expected pattern was observed in the two spontaneous speech tasks. Comparisons of variation in intensity/loudness, despite a much smaller sample size of participants with data on this variable, showed the expected pattern in adjusted tests.

**Discussion:**

Although values of each individual parameter across the five tasks tend to be highly correlated, it appears that different types of prompts for obtaining audio-recorded speech may in fact produce some differences across phonetic parameters. For example, whereas loudness appeared to be blunted equally across all of our tasks, variation in both pitch and F1 were blunted most obviously in the reading tasks, and reduced variation in F2 was most apparent in the two spontaneous speech tasks. Small sample size, no measures of negative symptoms in healthy controls and not controlling for patients’ medications are the main limitations of this work. Nonetheless, findings could represent a step toward developing new methods for measuring and tracking the severity of this specific negative symptom using acoustic phonetics parameters. Such work is relevant to other psychiatric and neurological disorders.

